# Inhibition of PPARα Induces Cell Cycle Arrest and Apoptosis, and Synergizes with Glycolysis Inhibition in Kidney Cancer Cells

**DOI:** 10.1371/journal.pone.0071115

**Published:** 2013-08-07

**Authors:** Omran Abu Aboud, Hiromi I. Wettersten, Robert H. Weiss

**Affiliations:** 1 Division of Nephrology, Department of Internal Medicine, University of California Davis, Davis, California, United States of America; 2 Comparative Pathology Graduate Group, University of California Davis, Davis, California, United States of America; 3 Cancer Center, University of California Davis, Davis, California, United States of America; 4 Medical Service, Sacramento VA Medical Center, Sacramento, California, United States of America; University of Kentucky College of Medicine, United States of America

## Abstract

Renal cell carcinoma (RCC) is the sixth most common cancer in the US. While RCC is highly metastatic, there are few therapeutics options available for patients with metastatic RCC, and progression-free survival of patients even with the newest targeted therapeutics is only up to two years. Thus, novel therapeutic targets for this disease are desperately needed. Based on our previous metabolomics studies showing alteration of peroxisome proliferator-activated receptor α (PPARα) related events in both RCC patient and xenograft mice materials, this pathway was further examined in the current study in the setting of RCC. PPARα is a nuclear receptor protein that functions as a transcription factor for genes including those encoding enzymes involved in energy metabolism; while PPARα has been reported to regulate tumor growth in several cancers, it has not been evaluated in RCC. A specific PPARα antagonist, GW6471, induced both apoptosis and cell cycle arrest at G0/G1 in VHL(+) and VHL(−) RCC cell lines (786-O and Caki-1) associated with attenuation of the cell cycle regulatory proteins c-Myc, Cyclin D1, and CDK4; this data was confirmed as specific to PPARα antagonism by siRNA methods. Interestingly, when glycolysis was blocked by several methods, the cytotoxicity of GW6471 was synergistically increased, suggesting a switch to fatty acid oxidation from glycolysis and providing an entirely novel therapeutic approach for RCC.

## Introduction

Renal cell carcinoma (RCC) is globally the 13th most common cancer, and one of the few cancers whose incidence is increasing for reasons that are not entirely clear but may be related to smoking and obesity (reviewed in [1] and [2]). Over the past several years, targeted therapies have become increasingly available and have shown considerable promise for the treatment of RCC and other malignancies; however, even with such therapies life expectancy is generally only extended by less than one year, owing to the development of drug resistance [3]. In light of the increasing number of patients presenting with late-stage disease and the prevalence of resistance to currently available drugs, new therapeutic targets are desperately needed. Identification of such targets could lead both to the design of new drugs and/or to the reevaluation of existing drugs for use in RCC patients.

The peroxisome proliferator-activated receptor α (PPARα) belongs to the steroid hormone receptor superfamily [4]. To date, three subtypes of PPAR (α, ß, and γ ) have been identified in many species including humans [5]. As occurs with other steroid hormone receptors, upon ligand activation, the PPARs heterodimerize with the retinoid X receptor (RXR), bind to the specific promoter sequence (the peroxisome proliferator response element or PPRE), and as a result trigger the expression of a variety of target genes [6] including those involved in glucose, lipid, and amino acid metabolism [7].

The PPARα receptors have an important, although likely pleiotropic given their multiple functions, role in malignancy. Whether they function as tumor suppressors or inducers in cancers is still uncertain; such functions may relate to cancer type and/or specific microenvironment of the tumor. While tumor suppression by PPARα has been reported in some cancers including melanoma [8] and glioblastoma [9], PPARα has also been found to lead to progression of tumor growth in other cancers including hepatocellular carcinoma [10] and breast cancer [11]. In our continuing study of kidney cancer using metabolomics methods, we found metabolic signatures of PPARα modulation in a human RCC cell (Caki-1) xenograft model across all three “matrices” (tissue, serum, and urine) [12]. Whether this finding is due to causality of PPARα activation in oncogenesis or whether it is simply a cancer “signature” was not determined in that study. Nevertheless, this finding led us to evaluate PPARα agonists and antagonists, for the first time, as potential RCC therapies.

We now show, using a specific PPARα antagonist as well as siRNA methods, that specific PPARα antagonism results in early cell cycle arrest as well as apoptosis in RCC cell lines. Furthermore, we provide evidence that when RCC cells are deprived of the glycolysis substrate, they become more sensitive to PPARα antagonists, suggesting that RCC cells alter their energy metabolism pathways under these conditions, and pointing to the feasibility of combination of PPARα antagonists and glycolysis inhibitor therapy for this disease.

## Materials and Methods

### Cell Lines

RCC cell lines, Caki-1, and 786-O were obtained from the American Type Culture Collection (Rockville, MD, USA), and the “normal human kidney” (NHK) cell line was obtained from Lonza (Basel, Switzerland). 786-O and Caki-1 cells were maintained in RPMI and NHK cells were maintained in DMEM, both supplemented with 10% FBS, 100 units/mL streptomycin, and 100 mg/mL penicillin. The cells were maintained at 5% CO_2_ and at 37°C.

### Materials

Formalin-fixed paraffin-embedded slides (hematoxylin eosin [H&E] staining and unstained) of archived RCC tissues were obtained from the UC Davis Department of Pathology after appropriate IRB approval. The PPARα agonist, WY14,643 (WY) and antagonist, GW6471 (GW) were dissolved in DMSO. WY, GW, DMSO, 2-Deoxy-D-glucose (2-DG), MTT solution, and mouse monoclonal anti-ß-actin antibody were obtained from Sigma (St. Louis, MO, USA). 2-DG was dissolved in water. Rabbit polyclonal anti-PARP antibody, mouse monoclonal anti-CDK4 antibody, rabbit polyclonal anti-cyclin D1 antibody, and rabbit polyclonal anti-c-Myc antibody were obtained from Cell Signaling Technology, Inc. (Beverly, MA, USA). Rabbit polyclonal anti-PPARα antibody was obtained from Abcam (Cambridge, MA, USA). Goat anti-mouse and goat anti-rabbit HRP conjugated IgG were obtained from Bio-Rad (Hercules, CA, USA). VECTASHIELD and DAB Peroxidase Substrate Kit, 3,3′-diaminobenzidine were purchased from Vector Laboratories (Burlingame, CA, USA). ECL Plus solution was obtained from Thermo Fisher Scientific (Waltham MA, USA). The PPARα and scrambled control siRNA were obtained from QIAGEN (Gaithersburg, MD, USA). Lipofectamine RNAiMAX was obtained from Invitrogen (Carlsbad, CA, USA).

### Immunohistochemistry

Human RCC (grades 1 and 4) and adjacent normal tissues were deparaffinized, pretreated in sodium citrate buffer, and blocked in the blocking buffer (5% normal goat serum and 0.3% Triton X-100 in PBS) for an hour at room temperature. After blocking, the slides were incubated with mouse monoclonal anti-PPARα antibody from Millipore (Billerica, MA) for overnight at 4°C. The slides were washed with TBST and incubated with 0.3% hydrogen peroxide in TBST for 15 minutes. The slides were washed with TBST, incubated with goat anti-mouse HRP conjugated IgG for two hours at room temperature. After washing, DAB Peroxidase Substrate Kit, 3,3′-diaminobenzidine was applied according to the manufacturer’s instructions. Hematoxylin was used for counter staining. The slides were coverslipped with VECTASHIELD.

### MTT Assay

Cell viability assay was performed as described previously [13]. Briefly, cells were plated in 96 well plates, and after the indicated treatments, the cells were incubated in MTT solution/media mixture. Then, the MTT solution was removed and the blue crystalline precipitate in each well was dissolved in DMSO. Visible absorbance of each well at 540 nm was quantified using a microplate reader.

### Cell Cycle Analysis

Cell cycle analysis was performed utilizing Muse™ Cell Analyzer from Millipore (Billerica, MA) following manufacturer’s instruction. Briefly, after the indicated treatments, the cells were washed with PBS and stained with propidium iodide (PI). After staining, the cells were processed for cell cycle analysis.

### Apoptosis Assay

Annexin V & Dead Cell Assay was performed utilizing Muse™ Cell Analyzer from Millipore (Billerica, MA) following manufacturer’s instruction. Briefly, after the indicated treatments, the cells were incubated with Annexin V and Dead Cell Reagent (7-AAD) and the events for dead, late apoptotic, early apoptotic, and live cells were counted.

### Immunoblotting

Immunoblotting was done as described previously [13]. Briefly, after the indicated treatments, the cells were washed with PBS, lysed in lysis buffer, and cell lysates were immunoblotted. The membranes were blocked in 5% nonfat dry milk for one hour at room temperature, incubated with indicated antibodies, and then probed with horseradish peroxidase tagged anti-mouse or anti-rabbit IgG antibodies. The signal was detected using ECL Plus solutions.

### siRNA Transfection

The indicated cells were plated in a six well plate for immunoblotting or T25 flasks for cell cycle analyses and apoptosis assays. After 24 hours, cell monolayers at approximately 75% confluency were subjected to siRNA transfection. The transfection mixture was prepared in Opti-MEM GlutaMax medium from Invitrogen (Carlsbad, CA, USA) with siRNA and Lipofectamine RNAiMAX according to the manufacturer’s protocol. The final concentration of siRNA added to the cells were 100 nM. The cells were cultured in the presence of transfection mixture for 24 h and the following day, the transfection mixture was replaced by fresh RPMI medium, and cell culture was pursued for an additional 48 hours. After the transfection, cells were collected for immunoblotting, cell cycle analysis, or apoptosis assay.

### Statistical Analysis

Comparisons of mean values were performed using the independent samples t-test. A p-value of <0.05 was considered significant.

## Results

### PPARα Shows Increased Expression in High Grade as Compared to Low Grade RCC

**Figure 1:**
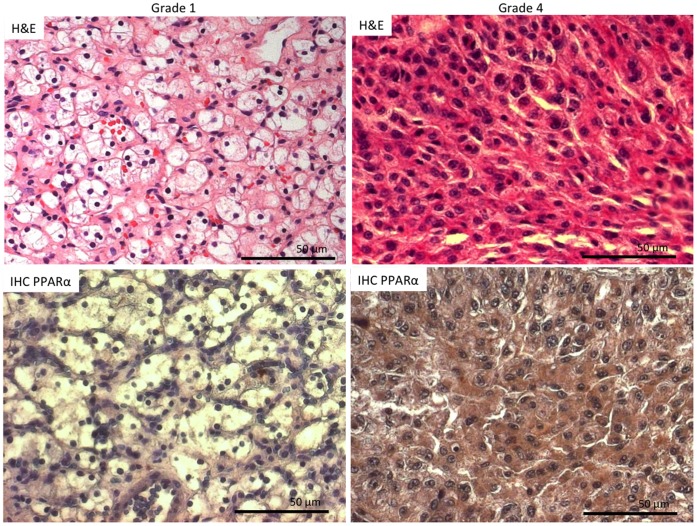
PPARα protein level was higher in grade 4 RCC tissues than grade 1 RCC tissues. RCC tumor tissues of different Fuhrman grades were prepared for immunohistochemistry as described in Materials and Methods and probed with PPARα antibody. The photomicrographs shown are representative of at least three patients for each group. Bar = 50 µm.

To begin to determine the relevance of PPARα in RCC, we first evaluated its protein levels in grade 1 and grade 4 RCC tissues by immunohistochemistry. Archived RCC tissues taken from nephrectomy samples were evaluated by immunohistochemistry with a specific PPARα antibody. RCC tissues with a histological diagnosis of Fuhrman grade 4 showed pronounced staining of PPARα while there was minimal staining of grade 1 tissues (Fig. 1). Of interest, the majority of the cytosol in grade 1 cells was comprised if a “clear” constituent, known to be glycogen and lipids, which did not stain with PPARα antibody [14].

### A Specific PPARα Antagonist, but not an Agonist, Attenuated RCC Cell Viability

**Figure 2:**
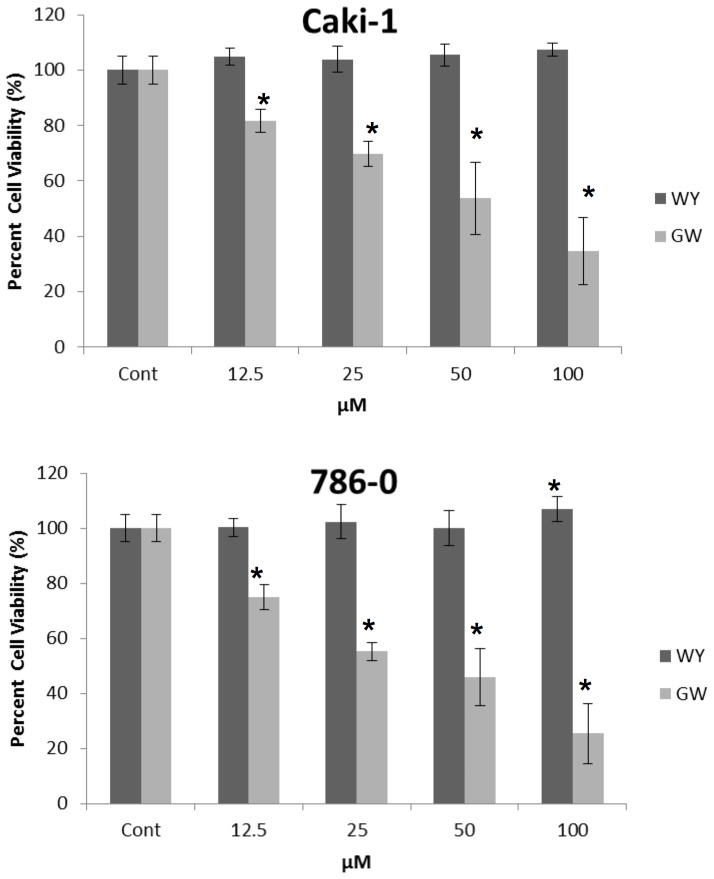
PPARα antagonist inhibited RCC and NHK cell viability. RCC cells (Caki-1 and 786-O) were treated with DMSO, WY14,643 (WY), or GW6471 (GW) at the indicated doses from 12.5 to 100 µM for 72 hours and a cell viability assay was performed as described in Materials & Methods. The data shown are representative of at least three repeats. *p<0.05 compared to DMSO. Error bars indicate standard deviation.

The increased levels of PPARα observed in high grade tissues provides little information concerning the functional status or signaling properties of this receptor. To begin to answer this question, we evaluated the functional role of PPARα on RCC cell viability by MTT assay. Both Caki-1 (VHL wild type) and 786-O (VHL mutated) cells were incubated separately with a specific PPARα agonist, WY14,643 [15], or a specific PPARα antagonist, GW6471 [16] at concentrations from 12.5 to 100 µM for 72 hours, and cell viability was assessed. While WY14,643 either had no affect on, or slightly increased, cell viability, GW6471 significantly and dose-dependently inhibited cell viability (up to approximately 80%) in both cell lines (Fig. 2).

### The PPARα Antagonist caused Both Cell Cycle Arrest and Apoptosis in both Cell Lines

**Figure 3:**
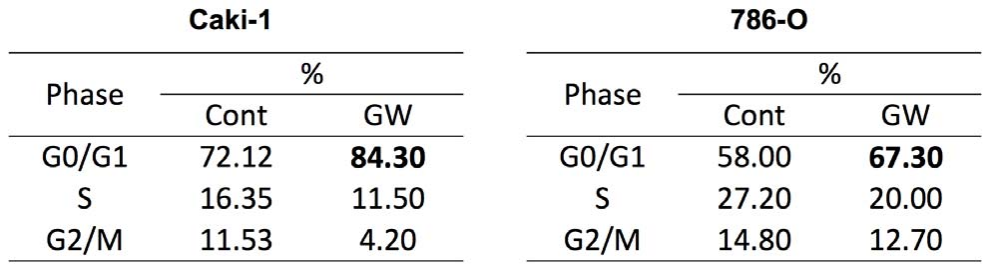
PPARα antagonist arrested cell cycle at G0/G1 phase and attenuated cell cycle related proteins. RCC cells (Caki-1 and 786-O) were treated with DMSO (Cont) or GW6471 (GW) 25 µM for 24 hours and cell cycle analysis was performed as described in Materials and Methods. The data shown are representative of at least three repeats.

The decreased cell viability observed after incubation of both RCC cell lines with the PPARα antagonist GW6471 could occur as a result of either decreased proliferation, induction of apoptosis, or both. To begin to answer this question, we first evaluated cell proliferation using flow cytometry methods. Both cell types were incubated with GW6471 or DMSO vehicle for 24 hours after which cell cycle analysis was performed. GW6471 arrested the cell cycle at the G0/G1 phase in both Caki-1 and 786-O cells (Fig. 3), suggesting that the MTT assay is, at least in part, indicating cell cycle arrest.

**Figure 4:**
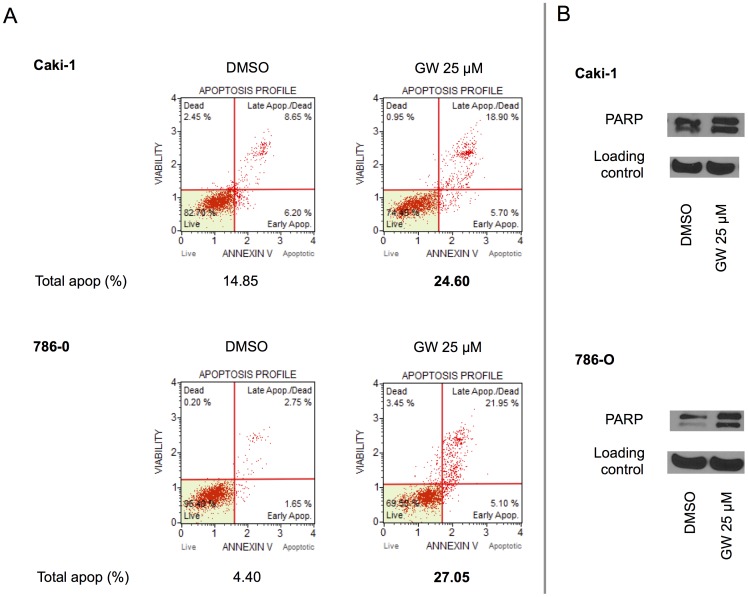
PPARα antagonist induced apoptosis in RCC cells. A. RCC cells (Caki-1 and 786-O) were treated with DMSO or GW6471 (GW) at the indicated doses for 24 hours and an annexin V-based apoptosis assay was performed as described in Materials and Methods. B. RCC cells (Caki-1 and 786-O) were treated with DMSO or GW6471 (GW) at the indicated doses for 24 hours and immunoblotting was performed as described in Materials and Methods. ß-actin was immunoblotted as a loading control. The data shown are representative of at least three repeats.

To determine whether PPARα antagonism resulted in apoptosis in addition to cell cycle arrest, we next evaluated annexin V staining under similar conditions as the above. After treatment of both cell lines with GW6471 or DMSO for 24 hours the cells were subjected to flow cytometery analysis after annexin V staining as described in Materials and Methods. As assessed by cell sorting, GW6471 increased the quantity of total apoptotic cells in both Caki-1 and 786-O cell lines (Fig. 4A). To confirm apoptosis under these conditions, PARP cleavage in the cells treated with GW6471 compared to DMSO was also assessed (Fig. 4B). Taken together, these data indicate that the reduced signal seen in the MTT assay after incubation of the cells with GW6471 is due to both cell cycle arrest and apoptosis induction.

**Figure 5:**
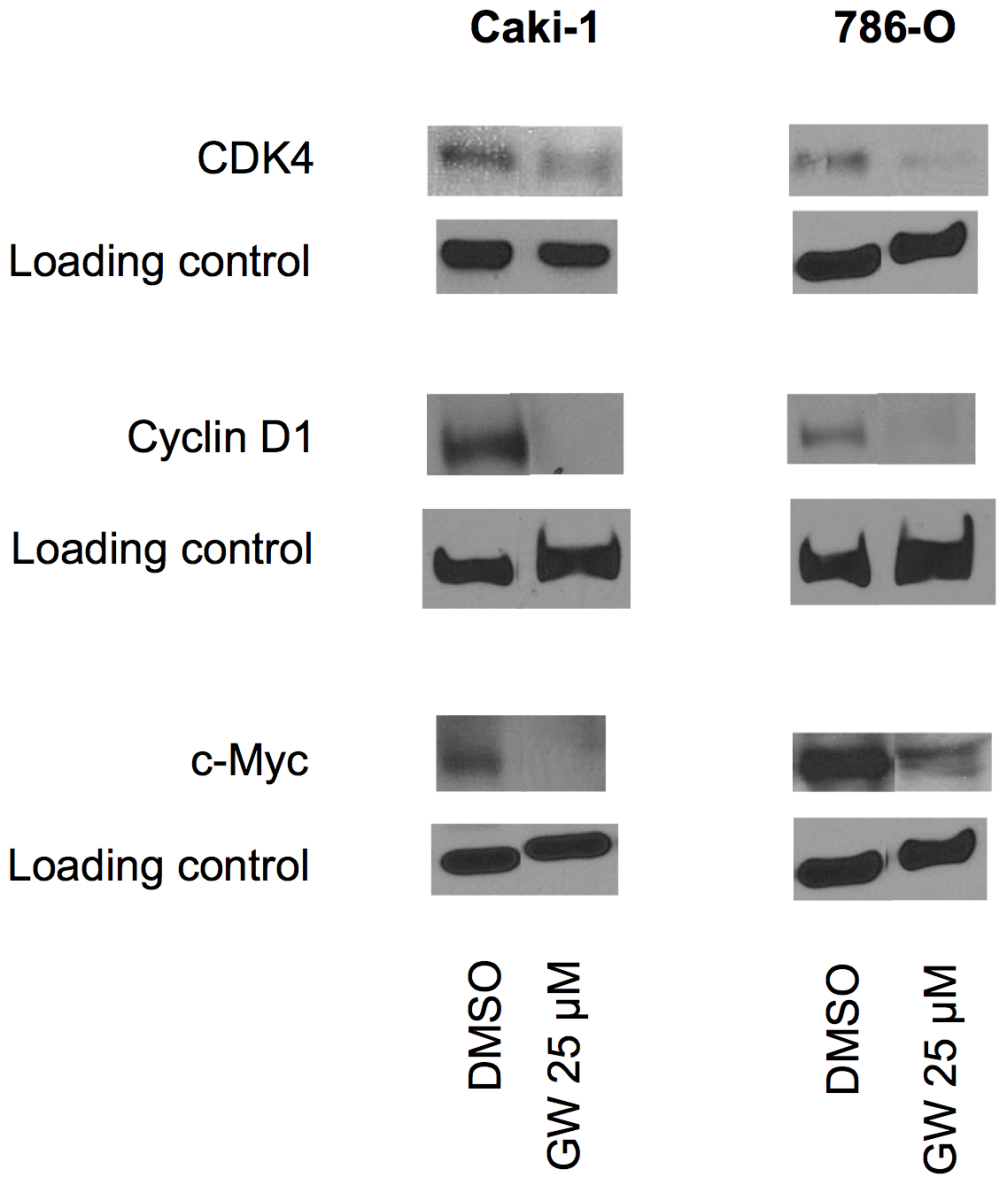
Levels of cell cycle and apoptosis relevant proteins were altered by PPARα Antagonist. RCC cells (Caki-1 and 786-O) were treated with DMSO (Cont) or GW6471 (GW) 25 µM for 24 hours and immunoblotting was performed as described in Materials and Methods. The pictures shown are representative of at least three patients for each group.

To further evaluate the mechanism of cell cycle arrest and apoptosis induction by GW6471, we measured levels of cell cycle and apoptosis relevant signaling proteins involved in regulating the G0/G1 checkpoint. Both cell lines were treated for 24 hour with GW6471, and then the cell lysate was immunoblotted with CDK4, cyclin D1, and c-Myc antibodies; all of these proteins were markedly decreased by the PPARα antagonist, supporting the observed G0/G1 arrest and suggesting a mechanism for same (Fig. 5).

### siRNA Transfections Confirm Specificity of the PPARα Inhibitor

**Figure 6:**
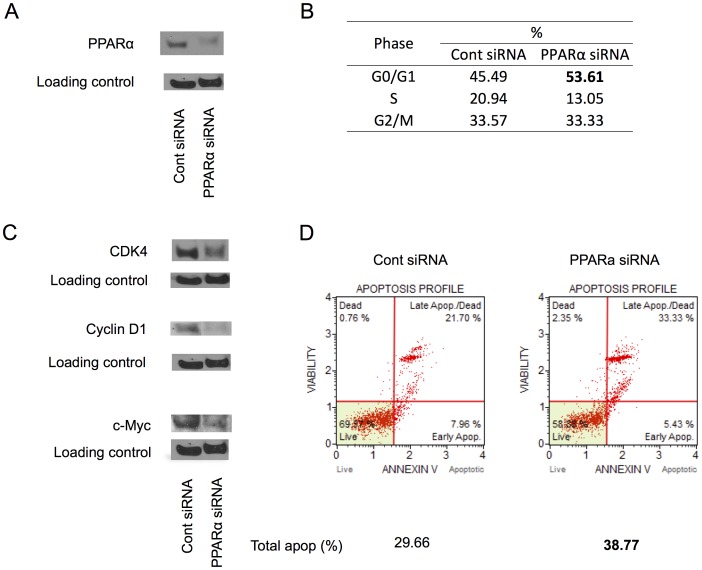
Downregulation of PPARα by siRNA transfection confirmed the antagonist’s on-target effects in Caki-1 cells. Caki-1 cells were transfected with control siRNA or PPARα siRNA at 100 nM for 72 hours and processed for immunoblotting, cell cycle analysis, and apoptosis assay as described in Materials and Methods. A. PPARα protein level was attenuated in the cells transfected with PPARα siRNA. B. PPARα siRNA transfection arrested cell cycle at G0/G1 phase. C. CDK4, cyclin D1, and c-Myc protein levels were attenuated in the cells transfected with PPARα siRNA. D. PPARα siRNA transfection induced apoptosis. The data shown are each representative of at least three repeats.

To confirm that the effects observed with GW6471 inhibition are specific to PPARα inhibition, we used an siRNA approach. Caki-1 cells were transiently transfected with a PPARα siRNA or scrambled sequence control siRNA as described in Materials and Methods. When compared to the control siRNA, PPARα siRNA attenuated protein levels of PPARα (Fig. 6A) similar to what was observed with GW6471 in this cell line (compare Fig. 6A to Fig. 5 left panel), confirming both efficacy of the siRNA and specificity of GW6471 towards PPARα. Several attempts were made to transfect 786-O cells with the identical siRNA but these were not successful.

To confirm that the cell cycle and apoptotic events observed with GW6471 incubation were in fact due to PPARα inhibition and not to off-target effects of the antagonist, we evaluated the cells under conditions of siRNA transfection parallel to GW6471 incubation. siRNA transfection of Caki-1 cells arrested the cell cycle at G0/G1 (Fig. 6B), attenuated protein levels of CDK4, cyclin D1, and c-Myc (Fig. 6C), and induced apoptosis (Fig. 6D) in an identical fashion to what was observed with GW6471 treatment, indicating that cell cycle arrest and attenuation of these proteins by GW6471 was in fact due to PPARα antagonism. Thus, the effects of GW6471 on the cell cycle, its regulatory proteins, and apoptosis resulted from specific PPARα antagonism.

### PPARα Antagonism and Glycolysis Inhibition Synergistically Attenuates RCC Cell Viability

**Figure 7:**
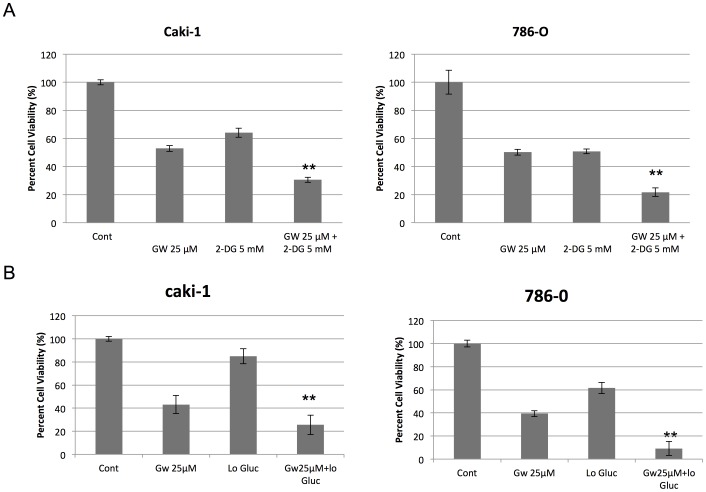
Glucose depletion synergized cytotoxicity of PPARα antagonist but not agonist. A. RCC cells (Caki-1 and 786-O) were treated with DMSO (Cont), GW6471 (GW) 25 µM, and/or 2-DG (5 mM) for 72 hours and cell viability assay was performed as described in Materials and Methods. B. RCC cells (Caki-1 and 786-O) were treated with DMSO (Cont), GW6471 (GW) 25 µM, low glucose media (Lo Glu), or GW6471 in low glucose for 72 hours and cell viability was assessed by an MTT assay as described in Materials and Methods. The data shown are representative of at least three repeats. **Synergistic effect compared to each treatment separately. Error bars indicate standard deviation.

Since PPARα has been known to activate fatty acid oxidation (FAO) and to decrease glucose utilization [17], we hypothesized that PPARα antagonism might cause the cells to decrease their reliance on FAO and thus be exquisitely dependent on glycolysis for their energy source; such a finding would suggest a novel approach for clinical utility of PPARα antagonists, especially with regard to RCC therapy. To evaluate this hypothesis, we treated RCC cells with GW6471 and/or the glycolysis inhibitor 2-DG and then measured cell viability. Under these conditions, there was a synergistic attenuation of cell viability with GW6471 and 2-DG. To confirm that 2-DG, which competes with glucose and hence attenuates cellular glycolysis, was causing the cells to decrease glucose utilization, we treated the cells with GW6471 grown in glucose depleted media. Both 2-DG treatment and glucose depletion sensitized RCC cells to PPARα antagonism (Fig. 7), suggesting basal dependence of RCC cells on PPARα-induced FAO such that the cells switch to glucose dependence when FAO is attenuated with PPARα inhibition. To evaluate potential differences in RCC vs. “normal” RTE cell lines, we performed parallel experiments in a “normal” human kidney cell line (NHK) obtained commercially and found similar changes (Figure S1). These data suggest that the dual inhibition of PPARα and glycolysis is a potential novel and powerful combination therapeutic approach for RCC.

## Discussion

RCC is the sixth most common cancer in the U.S. and is one of the few cancers whose incidence is currently increasing; the 5-y survival for patients with metastatic RCC is a dismal 26% (TNM Stage IV based on 2005 statistics) [18]. For the approximately one-third of patients who present with metastatic disease, there are several FDA-approved drugs available, among them the multi-kinase inhibitors (e.g. sorafenib and sunitinib) [19] and the mammalian target of rapamycin (mTOR) inhibitors [20]. Since progression-free survival even with these new drugs is a paltry one to two years, and because nearly all patients initially presenting with metastatic cancer succumb to their disease [21], it is essential to explore novel therapeutic approaches for patients with metastatic RCC.

PPARα is a ligand-activated transcription factor that belongs to the nuclear hormone receptor superfamily [22]. This receptor has been shown to stimulate fatty acid metabolism [17], to attenuate glycolysis [17], and to regulate tumorigenesis through promoting transcription of its target genes [23]–[28]. Despite extensive knowledge of the various targets of PPARα, the precise role of this receptor in regulation of cancer is still uncertain.

PPARα agonists have been shown to decrease the growth of melanoma, glioblastoma, and fibrosarcoma, and these effects have been associated with PPARα-induced inhibition of endothelial cell proliferation as well as PPARα-dependent down-regulation of cytochrome P450, resulting in inhibition of neoangiogenesis [29], [30]. On the other hand, activation of PPARα has been shown to increase proliferation in breast cancer cell lines [11]. Furthermore, long-term administration of PPARα agonists caused liver cancer in rodents [31], and *Pparα-*null mice were resistant to the hepatocarcinogenic effects of PPARα agonists [31], [32]. These data show that PPARα plays a pleiotropic role in cancer, but whether it functions as a tumor suppressor or an oncoprotein appears to be highly dependent on cancer type or even cell type.

As a basis for this study, we recently discovered a metabolic signature of RCC in mice xenografted with human RCC (Caki-1) cells [12]. Our study is supported by another in genitourinary cancers comparing mRNA and miRNA profiling, which showed evidenced of an enriched PPARα pathway in RCC but not in bladder cancer [33]. While these studies support an oncogenic effect of PPARα in RCC, the molecular mechanisms of tumorigenesis by PPARα and a potential therapeutic approach of PPARα inhibition have not been evaluated in RCC. In the present study we show for the first time that PPARα antagonism attenuates RCC cell growth through G0/G1 phase cell cycle arrest and the induction of apoptosis associated with decreased CDK4, cyclin D1, and c-Myc levels.

While there have been studies that show up-regulation of PPARα related metabolites [12] and genes [33] in RCC, no study has shown alteration of actual PPARα protein levels associated with tumorigenesis. In this study, we show increased PPARα levels in high grade RCC tissues vs. low grade tissues, suggesting that PPARα protein levels is associated with aggressiveness of RCC. Because PPARα regulates fatty acid oxidation (FAO) through its target gene transcription [34], and in light of the fact that grade dependent alterations of energy pathways (including FAO) proteins has been reported [35], it is possible that PPARα at least partially plays a role in aggressiveness and energy metabolism differences as a function of grade in RCC. This possibility is supported by the finding that low-grade RCC cells have more extensive clear cytosol, which consists of lipid and glycogen, than higher grade cells [14], [36].

To evaluate whether PPARα is a viable potential therapeutic target for advanced RCC, we analyzed the efficacy of PPARα antagonism utilizing a specific PPARα antagonist, GW6471, in RCC cell lines. Our data showed for the first time that such manipulation caused G0/G1 cell cycle arrest as well as induction of apoptosis. It is possible that these events are related to energy metabolism alterations which have been well studied in normal cells and in many diseases, including cardiovascular diseases and cancer, in which PPARα has been suggested as a therapeutic target [10], [17], [37], [38]. To our knowledge, ours is the first study to show cell cycle arrest by PPARα inhibition in RCC.

PPARα antagonism by both GW6471 and a specific siRNA showed decreases in c-Myc, cyclin D1, and CDK4. These findings are supported by a study which showed PPARα-dependent increases of c-Myc, cyclin D1, and CDK4 protein [39] by the PPARα agonist WY-14,643 in wild type mouse liver cells but not in the PPARα null cells. The cellular proto-oncogene, *c-myc,* is associated with a variety of human cancers and is strongly implicated in the control of cellular proliferation, programmed cell death, and differentiation [40]. PPARα has been shown to stabilize c-Myc protein through repression of the let-7c miRNA [41]. Thus, it is possible that attenuation of c-Myc protein in RCC cells by PPARα antagonism was through increased let-7c resulting in decreased stability of c-Myc. The cyclin D1/CDK4 complex promotes cell cycle progression through phosphorylation of its substrate including pRb (reviewed in [42]). Attenuation of c-Myc represses cyclin D1/CDK4 expression and activity at G1/S transition [43], [44]. These findings suggest that our observation that PPARα inhibition results in decreased c-Myc levels may account for the decrease in cyclin D1/CDK4 and thereby cause the observed cell cycle arrest at G0/G1 in RCC cells.

A significant finding from our study was that PPARα inhibition not only arrested cell cycle but also caused apoptosis in RCC cells. Interestingly, CDK4 inhibition has been reported to induce apoptosis [45] by causing translocation of RelA, the principal component of NFκB, from the cytoplasm to the nucleoplasm and then to the nucleolus resulting in repression of anti-apoptotic protein production including survivin. Because we observed profound CDK4 inhibition after PPARα antagonism, it is possible that PPARα inhibition-induced apoptosis was a result of CDK4 attenuation at least in RCC cells.

To further extend the potential of PPARα inhibition as a therapeutic approach, we sought combination treatments that increase efficacy of the PPARα antagonist. Since one of the roles of PPARα involves increasing FAO [10] as well as decreasing glycolysis at the transcriptional and functional levels leading to a decrease in pyruvate and lactate production [17], we hypothesized that PPARα antagonism may result in enhanced dependence of the cells on glycolysis due to the attenuation of FAO. Our finding that the efficacy of GW6471 was significantly higher in glucose-depleted media than the regular media further confirmed this supposition and further suggested that the synergistic effect of the PPARα antagonist and 2-DG combination treatment was specifically due to inhibition of glycolysis and not to off-target effects of 2-DG. Furthermore, this finding supports the future evaluation of dual therapeutics which could be used concurrently thereby attacking RCC at its Achilles heel of energy metabolism.

Our findings that NHK cells showed similar changes under these conditions should be tempered by several issues. First, the behavior of all cell lines, especially cells claimed to be “normal”, need to be evaluated in their in vivo context before firm conclusions can be drawn about their behavior, due to such issues as the stromal cell influence as well as cytokine release and other autocrine influences. Second, administration of GW6471 in a rabbit model (4 mg/kg as a bolus IV injection) [46] resulted in no gross changes in the kidney or alterations in urine output as compared to control animals after 5 hours (Christopher Lotz, personal communication).

In conclusion, we show here for the first time that (1) PPARα is upregulated in high grade RCC tissues compared to low grade tissues, (2) PPARα inhibition attenuates RCC cell viability through c-Myc, CDK4, and cyclin D1 decrease mediated cell cycle arrest and apoptosis induction, and (3) glycolysis inhibition synergizes with PPARα against cell viability. Taken together, these data suggest PPARα inhibition as a novel therapeutic approach for advanced RCC.

## Supporting Information

Figure S1 **Glucose depletion synergy with the PPARα antagonist occurs in primary normal human kidney epithelial (NHK) cells.** NHK cells were treated with 2-DG and subjected to no glucose media as described in Fig. 7. The data shown are representative of at least three repeats. **Synergistic effect compared to each treatment separately. Error bars indicate standard deviation.
(TIFF)
