# Supplementary figures and images for: Inhibition of PPARα Induces Cell Cycle Arrest and Apoptosis, and Synergizes with Glycolysis Inhibition in Kidney Cancer Cells
Source: PLoS One. 2013 Aug 7;8(8):e71115. doi: 10.1371/journal.pone.0071115 (PMC3737191; doi:10.1371/journal.pone.0071115)

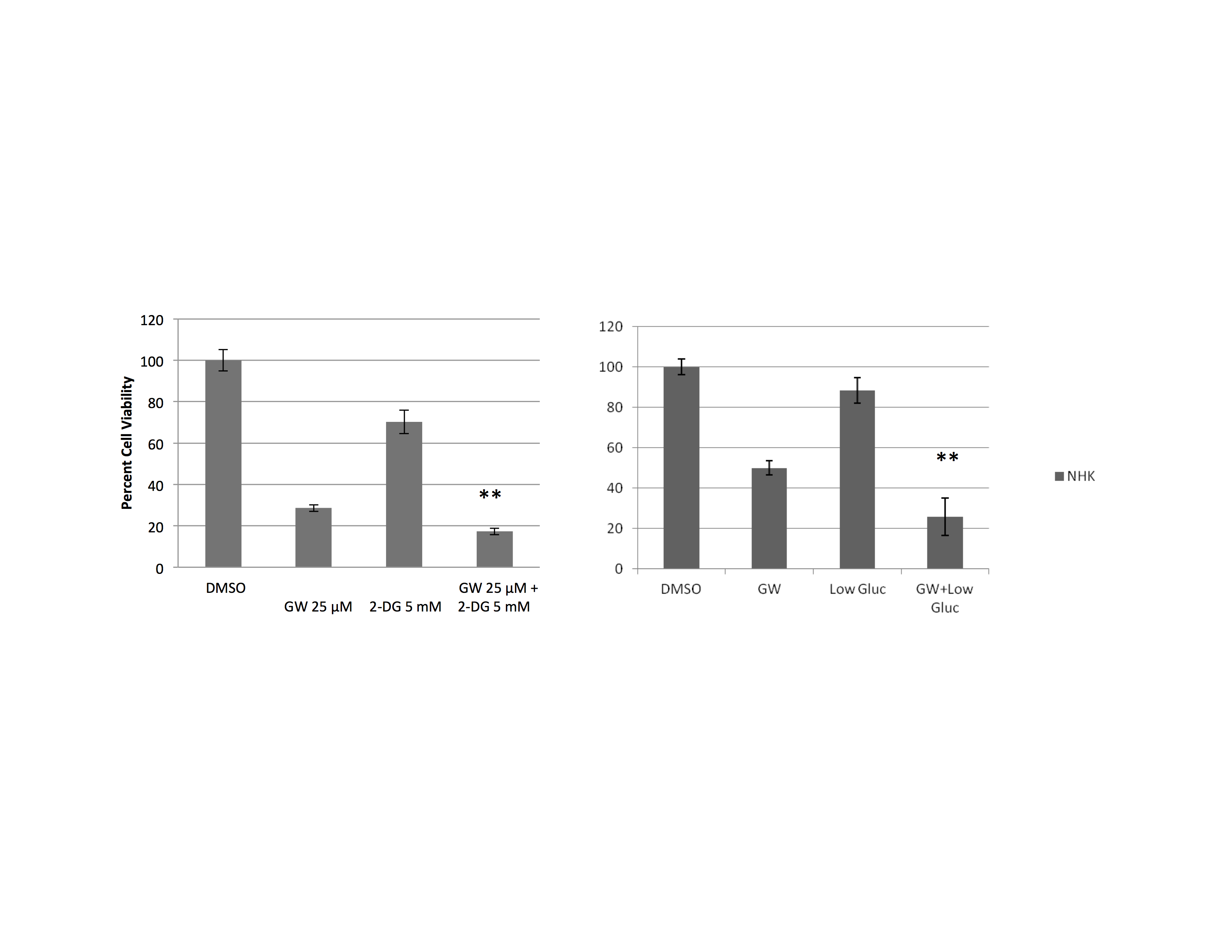

Supplement: Figure S1 — Glucose depletion synergy with the PPARα antagonist occurs in primary normal human kidney epithelial (NHK) cells. NHK cells were treated with 2-DG and subjected to no glucose media as described in Fig. 7. The data shown are representative of at least three repeats. **Synergistic effect compared to each treatment separately. Error bars indicate standard deviation. (TIFF) [file pone.0071115.s001.tiff]
